# An integrated approach to infer cross-talks between intracellular protein transport and signaling pathways

**DOI:** 10.1186/s12859-018-2036-2

**Published:** 2018-03-08

**Authors:** Kumar Parijat Tripathi, Marina Piccirillo, Mario Rosario Guarracino

**Affiliations:** Lab-GTP, High Performance Computing and Networking Institute, National Research Council, Via Pietro Castellino, 111, Naples, 80131 Italy

**Keywords:** Perturbation, Genetic Network, Secretory Pathway

## Abstract

**Background:**

The endomembrane system, known as secretory pathway, is responsible for the synthesis and transport of protein molecules in cells. Therefore, genes involved in the secretory pathway are essential for the cellular development and function. Recent scientific investigations show that ER and Golgi apparatus may provide a convenient drug target for cancer therapy. On the other hand, it is known that abundantly expressed genes in different cellular organelles share interconnected pathways and co-regulate each other activities. The cross-talks among these genes play an important role in signaling pathways, associated to the regulation of intracellular protein transport.

**Results:**

In the present study, we device an integrated approach to understand these complex interactions. We analyze gene perturbation expression profiles, reconstruct a directed gene interaction network and decipher the regulatory interactions among genes involved in protein transport signaling. In particular, we focus on expression signatures of genes involved in the secretory pathway of MCF7 breast cancer cell line. Furthermore, network biology analysis delineates these gene-centric cross-talks at the level of specific modules/sub-networks, corresponding to different signaling pathways.

**Conclusions:**

We elucidate the regulatory connections between genes constituting signaling pathways such as PI3K-Akt, Ras, Rap1, calcium, JAK-STAT, EFGR and FGFR signaling. Interestingly, we determine some key regulatory cross-talks between signaling pathways (PI3K-Akt signaling and Ras signaling pathway) and intracellular protein transport.

**Electronic supplementary material:**

The online version of this article (10.1186/s12859-018-2036-2) contains supplementary material, which is available to authorized users.

## Background

**Fig. 1:**
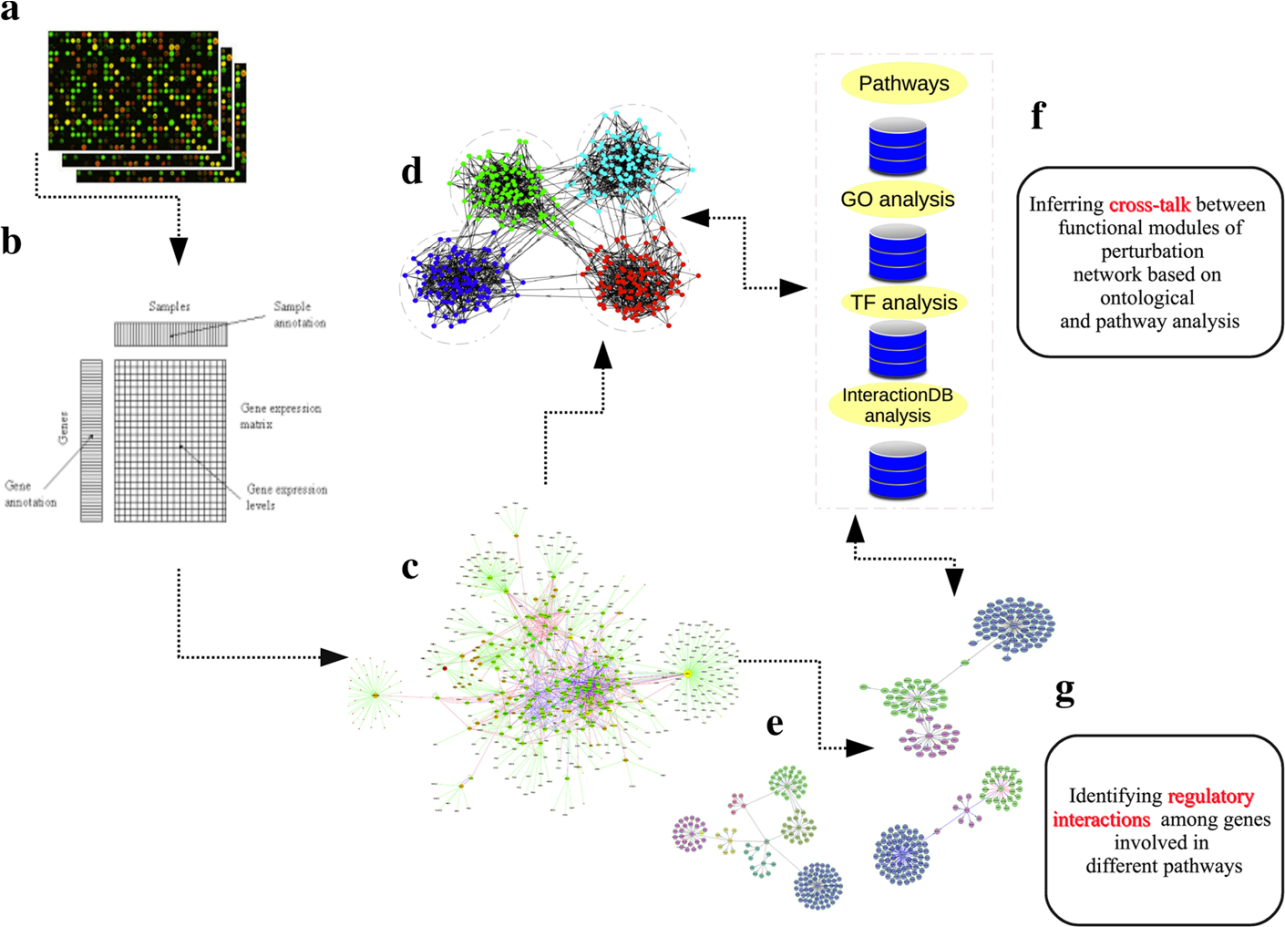
Schematic representation of the pipeline. **a** Perturbation experiments. **b** Perturbation expression profiles matrix. **c** Co-regulated gene-gene interaction network. **d** Identification and clustering of functional modules. **e** Identification of expression activated seb network (hotspot identification). **f** pathways and GO term enrichment analysis to infer cross-talk between different clusters. **g** Interaction database analysis along with functional annotation to infer regulatory patterns

The secretory pathway is composed of different organelles suspended in the cytoplasm. It includes rough endoplasmic reticulum (rough ER), ER exit sites (ERESs), the ER-to-Golgi intermediate compartment (ERGIC) and the Golgi complex cellular organelles, which have distinct functions in the transport of proteins to their final destination in the cell. Not only does the secretory pathway play an important role in proteins synthesis and delivery, but it also facilitates the proper folding and post-translational modifications of protein [1]. At present, we know that these organelles are able to interact dynamically with each other and play an important role in the establishment of cellular homeostasis; furthermore, the cross-talks between these inter cellular compartments are also required to maintain the structure and shape of the cell and for its survival [2]. Recent studies show all these cellular organelles within the secretory pathway are sensitive to stress conditions and capable to propagate the signaling for cell death [2]. Basically, signaling implies the conversion of mechanical or chemical stimuli directed towards the cell into a specific cellular response. In a general signaling pathway, a signal is received by the receptor molecules, which leads to a change in functioning and modulation of the cellular response driven by series of molecular interactions within the cellular boundary. These interactions include the activation and inhibition of numerous kinases and signaling molecules producing a complex inter dependent molecular cross-talks. To understand the complex relationship between signaling and secretory pathway in a broader perspective, it is important to study the genetic interactions within the cell and determine the gene regulatory network. Previously, researchers have been using correlation and gene co-expression based networks, to infer a genome wide representation of the complex functional organization of gene interaction networks [3]. These networks are predicted on the similarity of the gene expression profiles. However, these reconstructed gene networks are undirected, and therefore it is difficult to infer the causality relationship between two connected genes. The other caveat associated with co-expression network analysis regards the handling of large data sets, which limits the biological interpretation of the data [4, 5]. Though regression methods have been used to determine directed edges and to identify the set of genes having regulatory effects on their target, these methods are generally computational demanding and often limited to predict the set of genes regulated by transcription factors [6, 7]. Recently, gene perturbation studies have started playing an important role in directed gene networks reconstruction and in determining their reciprocal influence [8–11]. In the present work, we study the gene-gene interactions in MCF7 breast cancer cell line using an integrated approach (shown in Fig. 1) based on functional genomics and network analysis, derived from expression profiles of knocked-down or over-expressed genes within the secretory pathway. Signaling associated to and from protein transport machinery provides convenient therapeutic targets for drug development in cancer therapy [12]. Therefore, we try to decipher the direct and indirect genetic regulatory components of secretory pathway and their corresponding cross-talk with cellular signaling within the cell. Our goal is to understand the complex interactions among genes, constituting important signaling pathways with respect to protein transport in a cancer cells. Furthermore, we investigate the cause and disturbance in the delicate balance of cross-talks among these genes, which can lead to cancer progression. We try to highlight the interesting aspects of gene-gene interactions, which they could be as potential drug target for cancer therapies.

## Methods

### Data retrieval

To reconstruct the regulatory networks of secretory pathway we use the library of integrated network-based cellular signatures (LINCS) L1000, which contains more than a million gene expression profiles of perturbed human cancer cell lines (http://lincs.hms.harvard.edu). In more detail, it consists of 1328098 expression profiles of 22268 genes (Fig. 1a). A set of 978 genes, named *landmark genes*, were directly measured using a microarray technology. The remaining 21290 are *target genes*, whose expression has been inferred by a deep learning algorithm (D-GEX) trained on GEO data. Perturbation experiments were executed on different cell lines, at different time points, silencing or over-expressing genes, or treating cells with chemical compounds. For our work, we studied 3647 experiments in which genes have been knocked-down/over-expressed in MCF7 breast cancer cell line. Among the 2552 genes of the secretory pathway, only 591 were perturbed in L1000 dataset. For each perturbation experiment, we collect data for all the biological and technical replicates at two different time points (96H, 144H). We used the so called *level 3* dataset, which includes standardized gene expression profiles of directly measured landmark transcripts plus imputed genes (Fig. 1b). Finally, to map transcription factors and gene interactions information on the 591 perturbation experiments, we use the human transcription factors from AnimalTFDB 2.0 [13] and manually curated human signaling network data from Edwin Wang and associated group (http://www.cancer-systemsbiology.org/data-software). This signaling network dataset contains more than 6000 proteins and 63,000 relations. These relations represent activation, inhibition and physical interactions, which in turn describe complexes that play crucial roles in cell signaling.

#### Reconstruction of Regulatory Interactions

To obtain gene regulatory interactions and reconstruct gene network (Fig. 1c) from gene perturbation experiments, we developed a computational pipeline, which is divided into several steps. The z-scored perturbation expression profiles are represented as *Z*_*j*_∈*Z* with *j* = 1,…,*n*, in our case *n*=591. Each profile *Z*_*j*_ comprises *m*_*j*_ biological replicates (usually between 2 and 4), which are repeated measurements of biologically distinct samples and capture random biological variation [14]. Each biological replicate is the average of *q*_*k*_ technical replicates, which are repeated measurements of the same sample (usually between 4 and 6). A given perturbation experiment is represented as:

1 $$\begin{array}{@{}rcl@{}} {}P_{j,k,l} \ where \ j = {1,\ldots,n}; \ k = {1,\ldots,m_{j}}; \quad l = {1,\ldots,q_{k}} \end{array} $$

where *m*_*j*_ is the number of biological replicates in *j*-th profile, and *q*_*k*_ is the number of technical replicates for the *k*-th biological sample in the perturbation experiment. The mean biological sample $\overline {P}_{j,k}$ from the *q*_*k*_ technical replicates is calculated as:

2 $$\begin{array}{@{}rcl@{}} \overline{P}_{j,k} = \sum\limits_{l=1}^{q_{k}}P_{j,k,l}/q_{k} \end{array} $$

In the second step, we create a matrix with all biological replicates (averages), for which we calculate the first principal component, that is the linear combination of the biological replicates pointing in the direction of maximum variance. Before performing the Principal Component Analysis, we pre-processed the data to normalize their mean as follow:

3 $$\begin{array}{@{}rcl@{}} \overline{\overline{P}}_{j} = \sum\limits_{k=1}^{m_{j}}\overline{P}_{j,k}/m_{j} \end{array} $$

4 $$\begin{array}{@{}rcl@{}} Z_{j}=\left(\frac{\overline{P}_{j,1} - \overline{\overline{P}}_{j}}{\sigma_{j}}, \ldots, \frac{\overline{P}_{j,m_{j}} - \overline{\overline{P}}_{j}}{\sigma_{j}} \right) \quad \: j \: = {1,\ldots,n}, \end{array} $$

where *σ*_*j*_ is the standard deviation of $\overline {P}_{j,1},\ldots,\overline {P}_{j,m_{j}}$.

For each perturbation *j*, we select one biological replicate $\hat {Z}_{j}$, with maximum correlation with first principal component of *Z*_*j*_. Each column of the matrix $A=\left [\hat {Z}_{1}, \ldots, \hat {Z}_{n} \right ]$ represents the influence of perturbation on the expression values of all the genes in the experiments. Finally, for each perturbation, we selected only those genes, for which we observed a differential fold change ≥ 4 or ≤−4, in case of over expressed or under expressed genes respectively, in at least one biological replicate. Generally, any selection based solely on fold change is arbitrary and there is no right nor wrong threshold; but fold change (FC) cut-off of ≥ 2 or ≤−2, leads to look only at genes which vary widely among the other genes. For this reason in our work, to reduce the number of genes, we decided to use a most stringent FC, finally obtaining a list of 576 perturbed genes. The construction of the network is now straightforward, because the perturbation of a gene directly or indirectly affects the regulation of the others that have been detected as differentially expressed in that experiment. We applied a simplification algorithm to get rid of direct regulations introduced by the described reconstruction method [9].

### Network analysis

In this work we consider only gene interaction networks in which directed edges connecting two genes represent a biochemical process such as a reaction, transformation, interaction, activation or inhibition. We have not considered gene co-expression networks (GCN) in which the direction and type of co-expression relationships are not determined, because they are an undirected graphs where each node corresponds to a gene, and a pair of nodes is connected with an edge if they show a similar expression pattern. Therefore with the help of Cytoscape network visualization tool [15], we visualize the gene-gene interaction network from obtained regulatory interactions (Fig. 1c) and carry out directed network analysis using Network Analyzer [16]. We computed the topological parameters, such as number of nodes, edges and connected components for directed regulatory network. Further, we also computed the network diameter, radius, clustering coefficient, characteristic path length, betweenness and closeness, as well as the distributions of degrees, neighborhood connectivity and number of shared neighbors.

### Network hot-spot identification

In Cytoscape, the j-Active Modules plugin identifies expression activated sub-networks (Fig. 1e) from previously obtained molecular interaction network [17]. These sub-networks are highly connected components of the existing network, where the genes show similar significant expression changes in response to particular subsets of conditions (perturbations). The method uses a statistical approach to score sub-networks with a search algorithm for finding sub-networks with high score. The idea of finding these sub-networks is to determine functional modules represented by highly connected network regions with similar responses to experimental conditions. We run j-Active Modules on our gene interaction network in default mode, taking betweenness centrality and neighborhood connectivity as node attributes. In advanced parameter section, we set number of modules to 5 and overlap threshold to 0.8. Further, we employ *search* strategy to obtain high-scoring modules using local and greedy search.

### Reactome functional interaction (FI) network analysis

To study the pathways enrichment and network patterns in the sub-network with respect to signaling and intracellular protein transport, we use ReactomeFI-plugin [18] in Cytoscape, to integrate the Reactome database [19], and other tools such as Transcriptator [20] and Metabox library [21]. Taking a FDR cut-off value ≤0.05, we carry out pathway enrichment analysis for a set of genes in a given sub network, and investigate the functional relationships among genes in enriched pathways. With the help of this plugin, we first access the Reactome Functional Interaction (FI) network, and fetch FI indexing for all the nodes (genes) present in sub-network. Later, we build a FI sub-network based on a set of genes, query the FI data source for the underlying evidence for the interaction to construct modules by running a network clustering algorithm (spectral partition based network clustering) [22] and analyze these network modules of highly interacting groups of genes (Fig. 1d). Finally, we carried out functional enrichment analysis to annotate the modules, and expand the network by finding genes related to the experimental data set.

### Inferring gene regulatory interactions with respect to protein transport signaling

To understand the functional and regulatory relationship among genes in expression activated sub-networks, we use GeneMania plugin [23] (Fig. 1g). It extends the sub-networks by searching publicly available biological datasets to find related genes. These include protein-protein, protein-DNA and genetic interactions, pathways, reactions, gene and protein expression data, protein domains and phenotype screening profiles. Integration of physical interaction, genetic interaction, co-localization and pathway information related to the nodes present in the sub-networks, helps to show regulatory interactions among specific genes involved in signaling of protein transport.

### Extending regulatory interaction network through external resources to determine genetic cross-talks

By analyzing manually curated human signaling network data, we obtain further signaling interactions and introduce them to extend the regulatory interaction network already obtained from ReactomeFI network analysis. The signaling interaction data contain activation, inhibition and physical interactions. The physical relations represent complexes that play a role in cell signaling. Furthermore, we also map the transcription factors, gene ontology, and gene specific enriched reactome and KEGG pathways information on the reconstructed direct sub-network to infer direct physical and genetic interactions between perturbed genes and their effected components (Fig. 1f). We primarily focus on Ras and PI3K-Akt signaling pathways, to study and infer cross-talks among genetic components between them.

## Results and Discussion

### Biological function enrichment analysis of perturbed genes

We carried out Gene Ontology and pathway enrichment analysis of the complete list of perturbed genes, for which corresponding expression profiles are utilized in this study. It includes 576 unique perturbed genes which have regulatory effects on the other genes. DAVID and Transcriptator functional annotation tools are used to carry out enrichment analysis taking a multiple correction p-value cutoff ≤ 0.05. The complete list of perturbed genes is provided as Additional file 1. The functional term enrichment analysis suggests the role of these genes in intracellular protein transport such as exocytosis and endocytosis. As expected, the cellular components enrichment analysis suggests that most of these genes functions are localized in ER, Golgi apparatus, Golgi membrane, ER lumen, extracellular exosome, plasma membrane, trans-Golgi network, ER to Golgi transport vesicle membrane, endosome, endocytic vesicle membrane and lysosome etc. Similarly, biological process enrichment analysis shows biological processes related to regulation of intracellular protein transport such as exocytic and endocytic cellular mechanism, protein folding and modification process, as highly enriched terms. Some of these enriched biological functional terms are: positive regulation of protein phosphorylation, protein phosphorylation, protein glycosylation, ER to Golgi vesicle-mediated transport, retrograde vesicle-mediated transport, Golgi to ER, endocytosis, autophagy, Golgi organization, vesicle-mediated transport, sphingolipid biosynthetic process, ER unfolded protein response, ER calcium ion homeostasis, response to ER stress, IRE1-mediated unfolded protein response, lipoprotein biosynthetic process, protein autophosphorylation, chaperone-mediated protein folding, intrinsic apoptotic signaling pathway in response to ER stress and positive regulation of ERK1 and ERK2 cascade. The complete results of enrichment analysis is provided in Additional file 2.

### Regulatory interaction network from 591 gene perturbation expression data profile

**Fig. 2:**
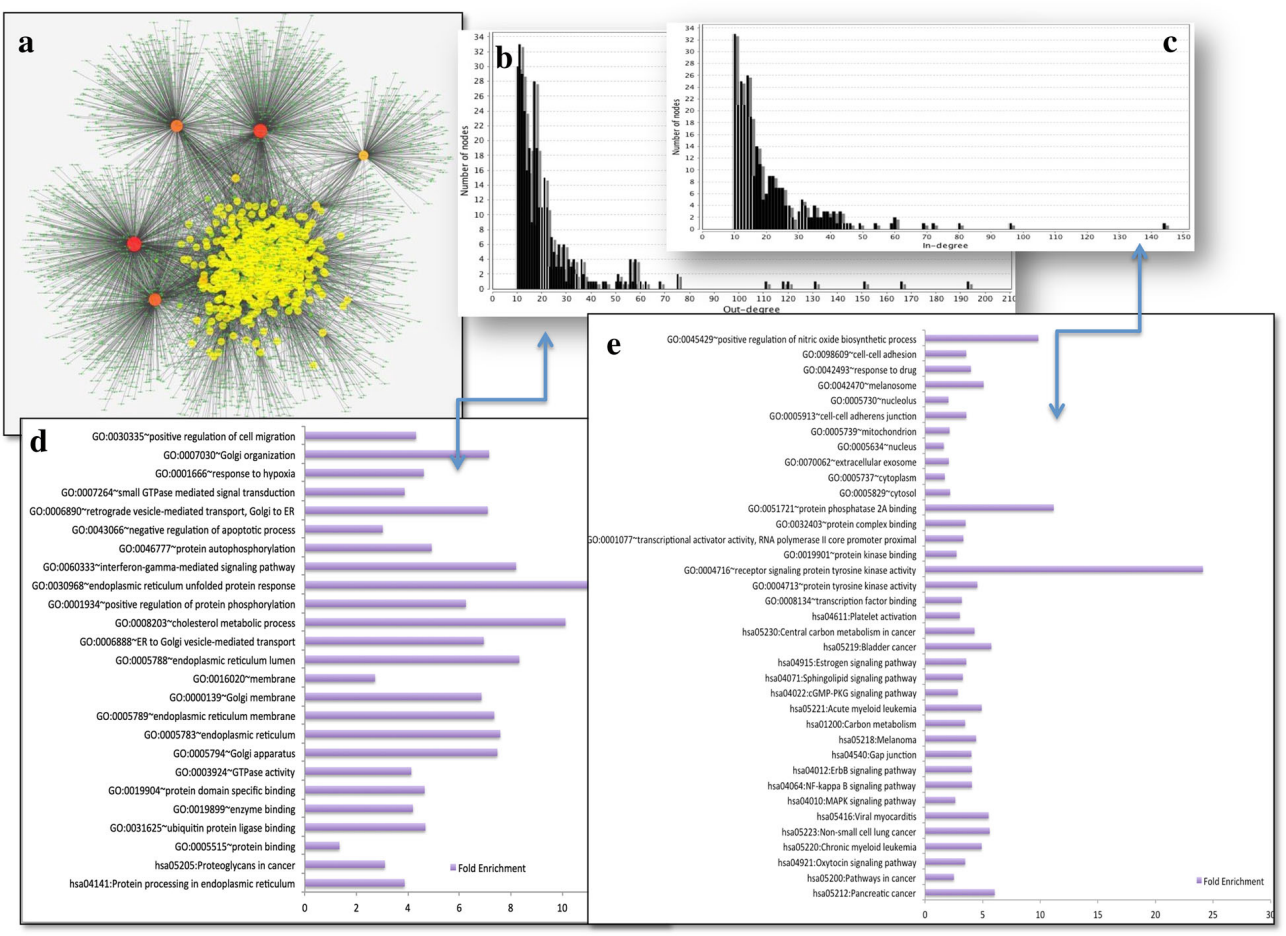
Regulatory interaction network. **a** Regulatory interaction network obtained from 591 perturbation experiments. The node color represents the degree of nodes; hub nodes are represented in red and yellow color. **b** out-degree distribution of nodes (perturbations). **c** in-degree distribution of nodes (effected genes). **d** Gene ontology enrichment analysis of perturbed genes. **e** Gene ontology enrichment analysis of effected genes

Using our pipeline we reconstructed a network based on regulatory interactions consisting of 4467 nodes and 12871 edges (Fig. 2a). The edges between the nodes in this network represent the 4 folds up or down regulation of affected genes in response to perturbation experiments. The characteristic path length of this network is 5.24 and average number of neighbors is 5.76. We calculate the following network statistics for all the constituting nodes in the network: topological coefficients, betweenness, closeness, distributions of degrees, neighborhood connectivity, average clustering coefficient and stress centrality. The table with all topological parameters for each node in the network is provided in Additional file 3. VHL shows a maximum out-degree of 923, which implies that its perturbation has a regulatory effect on the whole network. From the analysis of the network, we obtained 349 nodes having out-degree greater or equal to 10 (Fig. 2b). Among these 349 high out-degree nodes (represented in Fig. 2a, in darker color), there are 15 nodes which represent the maximum (≥ 100) of out-degrees, in other words, the perturbations in these genes have a significant effect on the transcriptional response. These nodes are represented in Table 1. Enriched Gene ontology terms associated with these genes are protein transport, localization, protein and lipid metabolism. Nodes (effected genes) with high in-degree’s (Fig. 2c) are highly enriched in kinase activity, transcriptional regulation, cell adhesion, RNA binding, protein binding, purine nucleotide binding, signal transduction, catalytic activities. The perturbed genes with high out-degrees in this directed network, are enriched in protein transport, localization and protein and lipid metabolism (Fig. 2d) and perturbation of these genes have direct and indirect regulatory effects on the elements of signal transduction, binding, transcriptional regulation and kinase activities (Fig. 2e). The complete functional enrichments are provided in the Additional file 4.

**Table 1:** 15 nodes with the maximum (≥ 100) of out-degrees

VHL	TOR1A	CSNK1D
CHEK2	STK16	POR
SP3	USP32	SRPRB
GPR107	RAF1	FEZ1
CFTR	PPAP2B	CRTAP

### Inferring gene regulatory components through the identification and analysis of expression activated (hotspot) sub-network

**Fig. 3:**
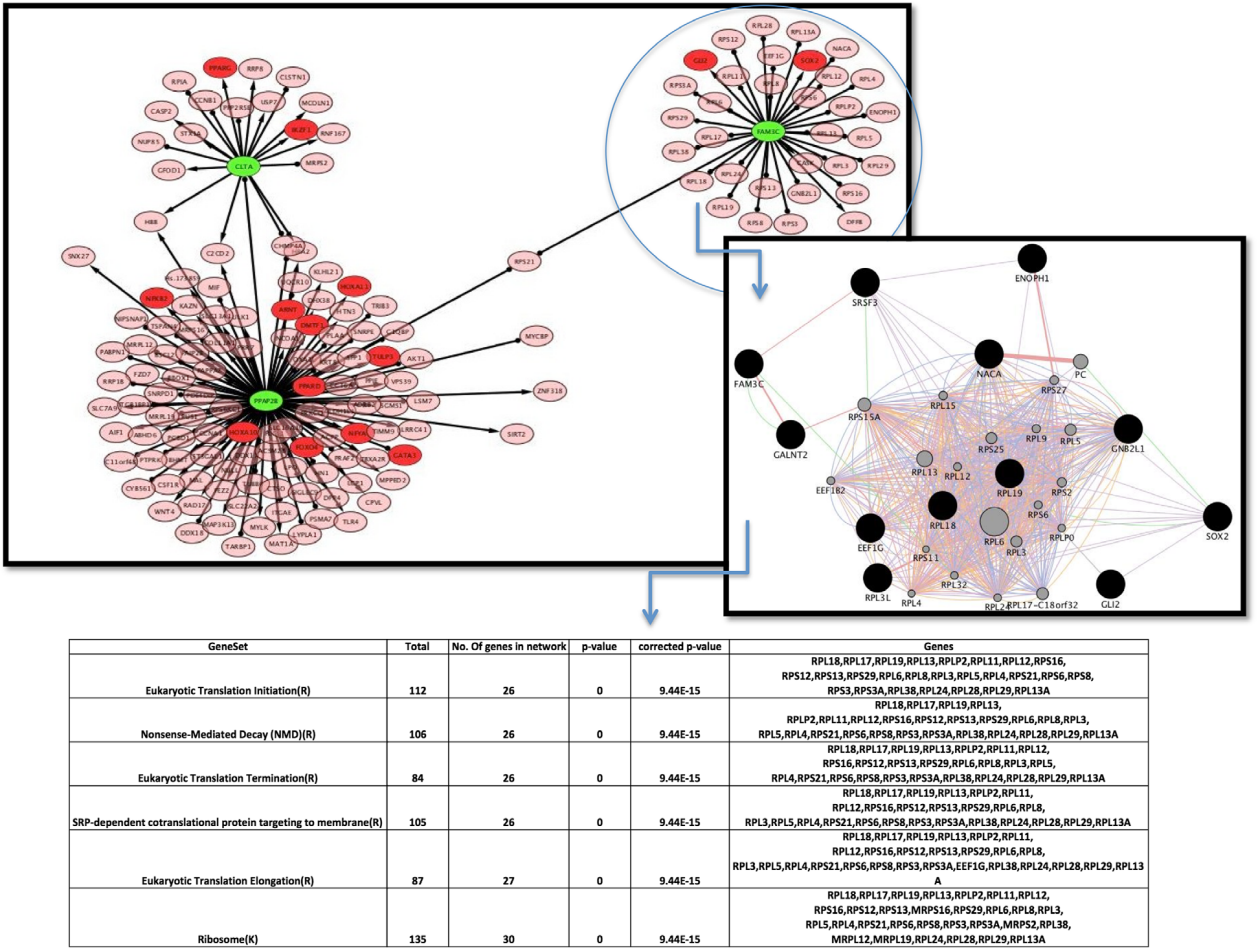
Network hot-spot Network hot-spot identified as gene regulatory network with respect to their regulatory interaction. Analysis of this network hot-spot through GeneMania analysis. Gene ontology (biological process) enrichment analysis for the network regulated by FAM3C perturbation. The edges with arrow signs represent the 4x increase in the expression of connected genes, while edges with dot represent the down-regulations. We map the information of perturbed genes (represented by green color) and transcription factor (in red color) on the network

With the help of j-Active plugin, we disaggregate the larger perturbation network into 5 smaller expression activated sub-network modules represented in Table 2. In the sub-networks, all the nodes are directly connected to the hub node/perturbed gene, and show similar significant changes (up or down-regulations) in expression in response to particular subsets of conditions (perturbations). For the sake of understanding the underlying gene-regulatory interaction within these sub networks, we selected the smallest module 5 for our study (Fig. 3). Firstly, we obtained the pathways, molecular function and biological processes enrichment analysis of module 5 sub-network using Reactome FI analysis. The genes are divided into three functional modules and are regulated by the perturbations of FAM3C, PPAP2B and CLTA respectively. Enrichment analysis shows that initiation, elongation and termination of translation processes, along with nonsense mediated decay are significantly enriched in genes regulated by FAM3C and perturbations of PPAP2B and CLTA do not have any effect on the regulation of these pathways. The perturbation of FAM3C may plays an important role in the eukaryotic translation pathways as well as nonsense mediated decay. Further, integration of co-expression, physical interaction, genetic interaction, co-localization and pathway information related to the nodes present in the sub-network, strengthen the relationship between FAM3C and its regulatory effects on the genes with respect to nonsense mediated decay. While many biological process, such as translation, RNA-metabolic process, cellular protein metabolic process, mesenchymal to epithelial transition, positive regulation of transcription, meiosis, negative regulation of interferon-gamma production, rRNA processing are not directly effected by the perturbation of FAM3C, they are regulated by the PPAP2B perturbation.

**Table 2:** The 5 modules obtained with j-Active Modules

Module	n of nodes	n of edges
1	3136	4424
2	375	573
3	334	364
4	225	231
5	159	162

### Cross-talks in signaling pathway

**Fig. 4:**
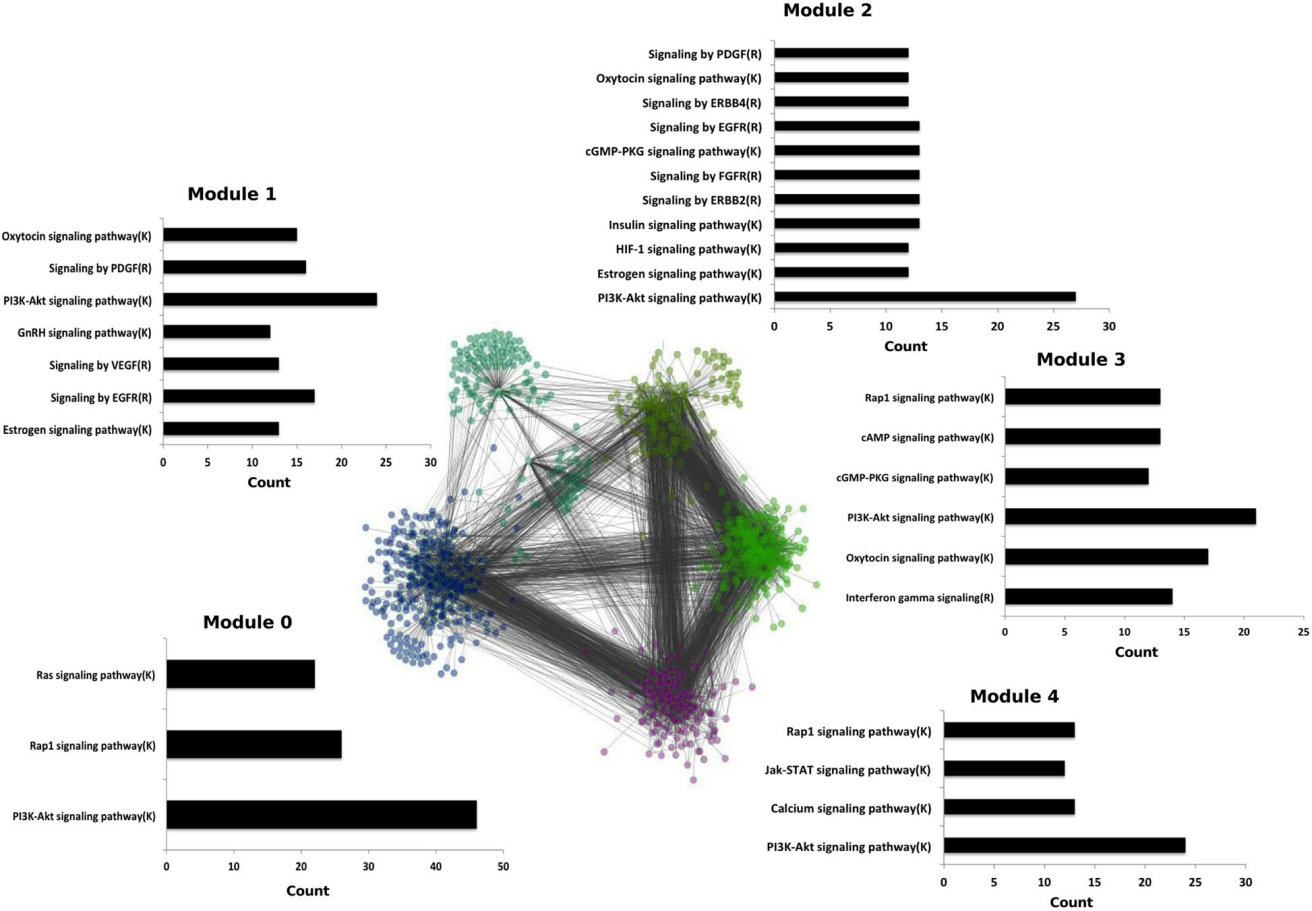
Clustering of regulatory network into 5 functional modules. Signaling pathway enrichment analysis in each functional module obtained from clustering of gene-gene interaction network. The color represents the functional module in clustered network. The blue color represents the module 0, light green represents module 1, khaki color represents module 2, bright green represents module 3 and in the last, module 4 is represented by violet color. The bar plot for each functional module shows the number of genes enriched in signaling pathways, by taking the cut-off for corrected *p*-value ≤ 0.05

To understand the complex gene-gene interaction network of cell signaling in the context of cellular protein transport and cancer progression, we carried out functional gene ontology and reactome pathways enrichment analysis for the constructed gene regulatory network. Based on the pathways enrichment, we extracted the sub-network consisting of gene/nodes involved in prominent signaling such as PI3K-Akt, RAP1, Ras pathway, calcium, P53 and MAPK signaling. Furthermore, we carried out the indexing of nodes with the help of ReactomeFI plugin and we clustered the sub-network into 5 functional modules based on gene ontology terms in biological process, molecular function, cellular components and reactome pathways enrichment analysis. The results are provided in the Additional file 5. Taking into account highly enriched signaling pathways (*p*-value ≤ 0.05) and cut-off for constituent nodes ≥ 12 in each module, we observed high intensity of cross-talks between signaling involved in protein transport (such as Ras signaling) and cancer progression (PI3K-Akt). Genes associated with PI3K-Akt signaling interacts with calcium signaling pathway. Considering module wise study, we notice that PI3K-Akt signaling is the most prominent and enriched signaling pathway, forming the core of each cluster (Fig. 4).

In cluster/module 0, PI3K-Akt interacts with Ras and Rap1 signaling pathways, while Ras pathway forms the major component of protein transport. Ras is a part of well described mitogen activated protein (MAP) kinase-Ras-Raf-MEK-ERK- pathway downstream initiated by receptor tyrosine kinase and integrins, leading to several cellular processes such proliferation, differentiation of cell, membrane genesis, protein synthesis and secretion and also has an intermediate effect on gene expression [1].

**Fig. 5:**
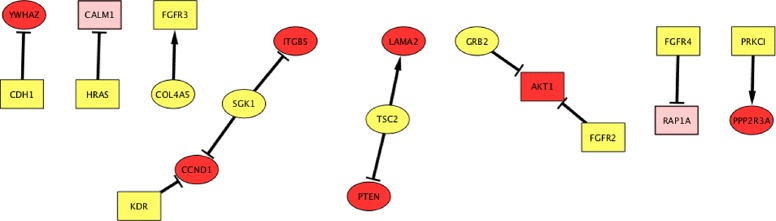
Interesting gene regulatory interactions with respect to PI3K-Akt signaling and Ras signaling pathway. PI3K-Akt signaling components are represented by oval shape. Genes involved in Ras signaling are represented by rectangular shape. Yellow color represent the perturbed genes involved in both signaling pathway. In this figure, we have shown the PI3K-Akt enriched genes in red color, while the genes enriched in Ras pathway are represented by rectangular shape. The yellow color represents the genes which undergoes perturbation experiments to obtain the gene-gene regulatory network. The edges with arrow signs represent the four fold increase in the expression of target/effected genes, while edges with dot represent the four fold down-regulation

In cluster/module1, cluster/module2 and cluster/module3, we observe the interactions between PI3K-Akt and kinase signaling such as *ERBB2*, *EGFR* and *ERBB4*. In the last module/cluster 4, enrichment of PI3K-Akt signaling, JAK-STAT and calcium signaling suggests activation of PI3K-Akt signaling through both calcium and stress-activated protein Jun kinases and vice versa. In the past, researchers portrays the role of intracellular Ca2+ and disturbances in its cellular concentration, with respect to tumor initiation, angiogenesis, progression and metastasis in the normal cells [24]. To delve further into the relationship between Ras and PI3K-Akt signaling, we extracted the constituent genes/nodes from regulatory interactions within module 0, and we observed interesting relationship among them, as shown in Table 3. The regulatory interactions among these genes (Fig. 5) show that important constituent genes in PI3K-Akt signaling are regulated by the genetic components of Ras signaling pathway. In some cases, common genes regulate both pathways, exhibiting higher level of cross-talk between them. It is worth noting that perturbation of *CDH1* leads to the 4 fold decrease in the expression of *YWHAZ* gene, which is a member of the 14-3-3 protein family and a central hub protein for many signal transduction pathways. YWHAZ gene regulates apoptotic pathways critical to cell survival and plays a key role in a number of cancers and neuro-degenerative diseases [25]. This gene is a well-known target for cancer therapy (14-3-3 zeta as novel molecular target for cancer therapy). Hence *CDH1* could be a potential gene as a molecular target for cancer therapy.

**Table 3:** Genes enriched in PI3K-Akt and Ras signaling

Signaling pathway	Genes
PI3K-Akt	YWHAZ,COL4A5,SGK1,ITGB5,TSC2,LAMA2,
	PTEN,GRB2,PP2R3A
Ras signaling pathway	CDH1,HRAS,CALM1,FGFR3,KDR,AKT1,FGFR4,
	Rap1A,PRKC1
PI3k-AKT and Ras signaling	HRAS,KDR,FGFR3,AKT1,FGFR2,FGFR4

All these genetic regulatory interactions might provide a viable target for cancer drug therapy. From our results, we observed that PTEN is heavily down-regulated by the perturbation of *TSC2* gene. Both the genes play an important role in PI3K-Akt signaling. *PTEN* gene is a tumor suppressor [26], and mutation in this gene leads to cancer developments. *TSC2* mutations lead to tuberous sclerosis, and its gene products is supposedly a tumor suppressor [27]. From this information, we can infer that perturbation of TSC2 gene plays an important role in increasing cancer risk in muscular dystrophy, as it regulates LAMA2 gene. Genetic mutations in LAMA2 genes have their implication in a severe form of muscular dystrophy [28]. *TSC2, LAMA2* and *PTEN* interactions could be useful to study a potential drug therapy for cancer as well as muscular dystrophy. Similarly *CCND1* amplification and its protein expression is strongly correlated with breast cancer [29], the perturbation of KDR gene, which is a type III receptor tyrosine kinase involved in Ras pathway, down-regulates the *CCND1* expression and controls its amplification with respect to cancer.

From the regulatory interaction network analysis, we infer that genetic perturbations involved in protein transport have profound effects on the signal transduction, and transcriptional regulation activities of the cell. We also carried out functional analysis of the nodes with high value of betweenness centrality, as these nodes do play an important role in bridging between the sub-networks and hub nodes. The results show several enriched pathway: ER-nucleus signaling pathway (GO:0006984), cellular response to topologically incorrect protein (GO:0035967), response to topologically incorrect protein (GO:0035966), response to unfolded protein (GO:0006986) with significant *p*-value ≤ 0.05. Furthermore, functional enrichment analysis of the nodes with respect to out-degree, in-degree and betweenness centrality, helps us to understand the underlying cross-talk between protein transport, localization on the signal transduction and transcriptional rewiring and their mutual effects on protein folding. In our regulatory network, we find some interesting and significantly enriched signaling pathways, such as PI3K-Akt, Ras, Rap1, calcium, JAK-STAT, EFGR and FGFR signaling. In recent years, researchers observed the role of PI3K-Akt signaling in cancer progression, which is basically a disturbance in the balance of cell division and growth with respect to programmed cell death. This particular signaling pathway is disturbed in many human cancer and not only does it play a major role in tumor development, but also in its potential response to the treatment [30]. In our results, we observe that PI3K-Akt signaling interacts with several kinases, such as ERBB2, EGFR and ERBB4. These kinases are known to play an important role in a very aggressive form of breast cancer [31]. This kind of signaling leads to a characteristic behavior of cancer cells such as uncontrolled proliferation, resistance to apoptosis and increased motility. Apart from this, PI3K-Akt signaling shares interactions with platelets and fibroblast growth factors signaling pathways, which play a very important part in cell growth regulation, proliferation, survival, differentiation and angiogenesis [31]. Most of these pathways are involved in the normal deployment of protein transport but also have a potential role in activating both upstream and downstream important signaling pathways. Some of these functions are cell proliferation, differentiation, membrane biogenesis, inflammation protein syntheses, cell migration and gene expression regulation. In a broader sense, all these signaling networks comprise a fine tuning balance for cellular function. Any disturbance in such a balance leads to negative signaling cascades and has a deteriorating effect on cell functioning, possible leading to cancer progression.

### Cross-talks between intracellular protein transport and signaling pathways

**Fig. 6:**
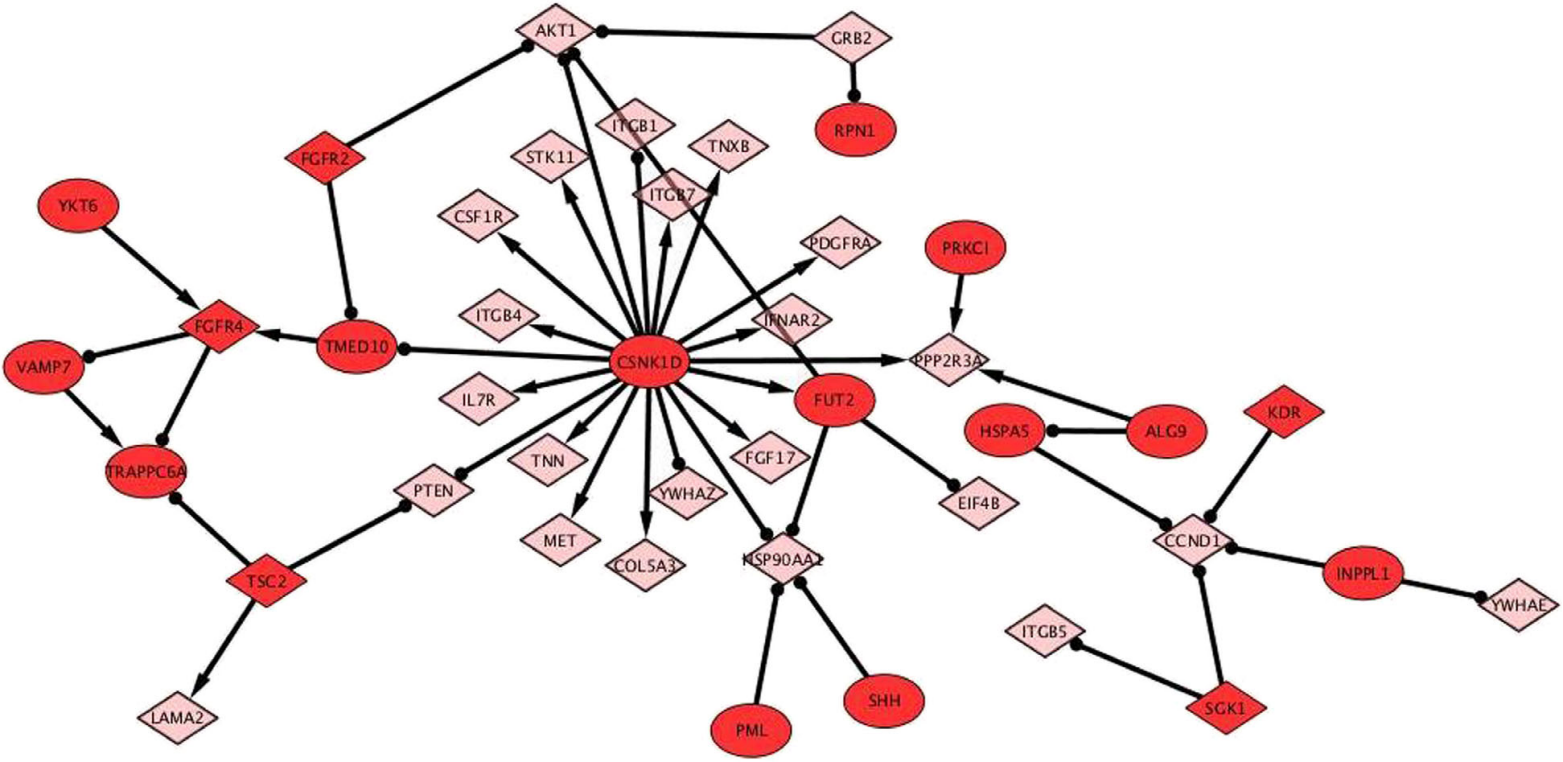
Regulatory interactions between PI3K-Akt signaling and intracellular protein transport. Genes enriched in PI3K-Akt signaling pathway are represented by diamond shape in pink color such as IL7R,TNN, ITGB4, CSF1R, STK11, ITGB1, ITGB7, FGF17, COL5A3, PDGFRA, PP2R3A, AKT1, HSP90AA, CCND1 and PTEN and YWHAZ. These genes are regulated by the perturbation in CSNK1D, TSC2, PML, SHH, KDR and SGK1 gene represented by oval shape in red color. Out of these perturbed genes, KDR, SGK1 and TSC2 also plays an important role in PI3K-Akt signaling; and they are represented by diamond shape and in red color. The edges with arrow signs represent the four fold increase in the expression of target/effected genes, while edges with dot represent the four fold down-regulation

In addition to study the cross-talks between different signaling pathways in intracellular protein transport, we also infer the regulatory effects of signaling pathways on intracellular protein trafficking mechanism related to exocytic and endocytic pathways [32]. Through exocytic pathway, protein cargo moves from ER, via Golgi apparatus, to the plasma membrane. During this movement, it also undergoes to a modification by the addition of sugar and lipids. On the other hand, moving through this forward exocytic pathway via ER-Golgi-plasma membrane compartments, the protein cargo has to be retrieved back to its original compartment in a reverse direction, to maintain the compartment identity. This backward movement of protein cargo from plasma membrane to Golgi to ER compartment is known as *retrograde protein transport*. There is also an endocytic pathway, through which cargo is internalized from the cell milieu. The best characterized endocytic pathway proceeds from clathrin coated vesicles through early and late endosomes to lysosomes. The lysosomes is a major degradation site for internalized cargo and cellular membrane proteins [32]. In our results, we observed regulatory interactions among genes involved in intracellular protein transport and PI3K-Akt, Ras, MAPK, interferon and calcium signaling. In the modules study, we specifically focus on PI3K-Akt and Ras signaling pathways and their regulatory interactions with intracellular protein transport components in MCF-7 cell line. In the previously described module 0, as shown in Fig. 6, genes enriched in PI3K-Akt signaling pathway such as IL7R,TNN, ITGB4, CSF1R, STK11, ITGB1, ITGB7, FGF17, COL5A3, PDGFRA, PP2R3A, AKT1, HSP90AA, CCND1 and PTEN and YWHAZ are regulated by the perturbations in CSNK1D, TSC2, PML, SHH, KDR and SGK1 genes. Out of these perturbed genes, KDR,SGK1 and TSC2 also play an important role in PI3K-Akt signaling [33, 34]. The CSNK1D gene is involved in protein phosphorylation process, endocytosis and Golgi organization [35]. The result agrees with the fact that PI3K-Akt is a signal transduction pathway, which helps in cell survival, growth, proliferation, cell migration and angiogenesis. The key proteins are PI3K (phosphatidylinositol 3-kinase) and AKT (Protein Kinase B). It is interesting to observe that CSNK1D perturbation positively regulates most of the components of PI3K-Akt signaling except it down-regulates the PTEN gene. The PTEN (phosphatase and tensin homolog) gene is a major antagonist of PI3K activity [36]. It is a tumor suppressor gene and often mutated or lost in cancer cells. Additionally, CSNK1D perturbation also down-regulate YWHAZ gene, which is also a major regulator of apoptotic pathways and plays an important role in cell survival [25, 37, 38]. Being a constituent of PI3K-Akt signaling, TSC2 gene also contributes to endocytosis, and when perturbed, down-regulates the PTEN gene. PML and SHH genes, which are associated with ER calcium ion homeostasis and endocytosis process respectively, along with CSNK1D gene, down-regulate HSP90AA2 (Heat shock protein 90kDa alpha (cytosolic), class A member 2) gene. HSP90AA2 is a heat shock gene, generally expressed to combat a stressful situation and whose protein product functions as chaperon by stabilizing new proteins to ensure correct folding [39]. In summary, we observe that the perturbation in genes involved in protein phosphorylation, endocytosis, Golgi organization and calcium ion homeostasis in ER, have a stronger effect in the activation of PI3K-Akt signaling, and in the down regulation of PTEN, YWHAZ and HSP90AA2 genes which are important for the normal functioning of the cell.

**Fig. 7:**
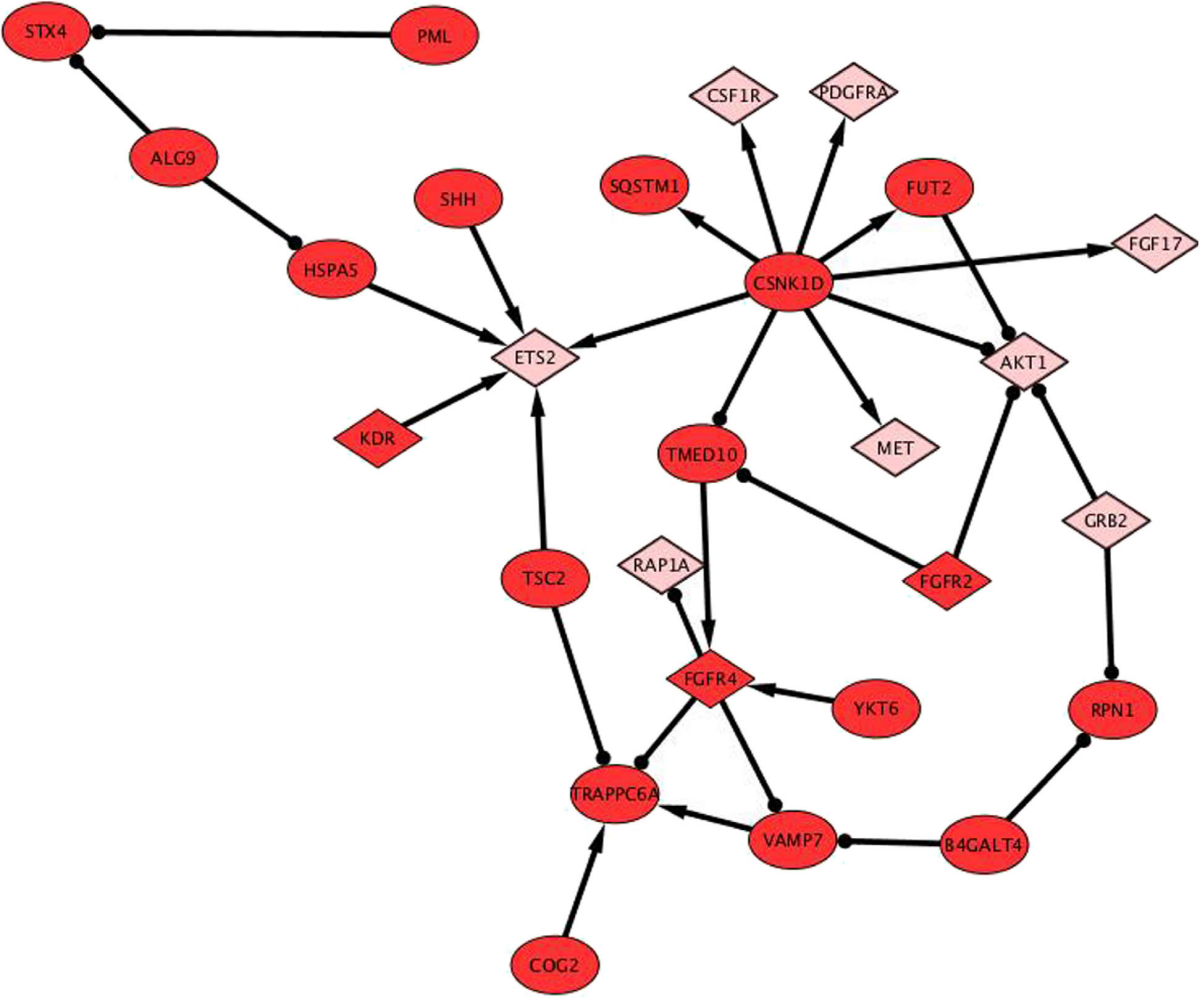
Ras signaling cross-talk protein transport. The cross regulatory interactions between the components of Ras (represented by diamond shape and in pink color) and intracellular pathways represented by oval shape and in red color. Constituent genes of Ras pathway such as CSF1R, PDGFRA, FGF17, FGFR2, FGFR4, RAP1A, GRB2 and KDR are either regulated by the components of intracellular protein transport or they also regulate each other variably. Some of the components of Ras pathway such as KDR, FGFR4, FGFR2 are also involved in intracellular protein transport mechanism. The edges with arrow signs represent the four fold increase in the expression of target/effected genes, while edges with dot represent the four fold down-regulation

Similarly, we also observe the cross-talks between Ras signaling pathways and intracellular protein transport mechanism. The cross regulatory interactions between the components of Ras and intracellular protein transport pathways are depicted in resultant (Fig. 7). We observe constituent genes of Ras pathway such as CSF1R, PDGFRA, FGF17 [40], FGFR2, FGFR4, RAP1A [41], GRB2 [42] and KDR are either regulated by the components of intracellular protein transport or they also regulate each other. CSF1R (Colony stimulating factor 1 receptor (CSF1R)) is a receptor for a cytokine called colony stimulating factor 1. PDGFRA (platelet-derived growth factor receptor A) encodes a typical receptor tyrosine kinase, which binds to platelets derived growth factors and plays an active role in initiating cell signaling pathways responsible for cellular growth and differentiation [43–45]. Both of them are positively regulated by the perturbation in CSNK1D gene [46]. The CSNK1D gene is involved in endocytosis, Golgi organization,positive regulation of protein phosphorylation,protein phosphorylation. Some of the components of Ras pathway such as KDR [47], FGFR4 [48], FGFR2 [48, 49] are also involved in intracellular protein transport mechanism [50]. KDR which is a type III receptor tyrosine kinase, also known as vascular endothelial growth factor receptor 2 (VEGFR-2), is also involved in positive regulation of protein phosphorylation [50]. Perturbation in KDR, affects the ETS2 gene in Ras pathway. Fibroblast growth factor receptor 2 (FGFR2) and Fibroblast growth factor receptor 4 (FGFR4) are members of the fibroblast growth factor receptor family. These receptors signal by binding to their ligand and dimerisation and initiate a cascade of intracellular signals. These signals are involved in cell division, growth and differentiation [51, 52]. Perturbation in FGFR2, down-regulates TMED10 and AKT1. TMED10 is involved in ER to Golgi vesicle-mediated transport, retrograde vesicle-mediated transport, Golgi to ER, Golgi organization. While AKT1 is a serine-threonine protein kinase, activation of this gene phosphorylates and inactivates components of the apoptotic machinery [53–55]. Perturbation in FGFR4 gene, down-regulates VAMP7 [56, 57] and RAP1A expression [58], which are involved in ER to Golgi vesicle-mediated transport, endocytosis, vesicle-mediated transport and Ras Pathway respectively. The results of all the regulatory interactions and subsequent enriched pathways are provided in the Additional file 6.

## Conclusions

In this work, we develop a computational integrated pipeline to analyze genes perturbation experimental data and uncover the regulatory interactions among genes. We use functional genomics and network biology approach to create a directed network, where nodes represent the perturbed and impacted genes, while direct edges represents the positive and negative regulatory effects of perturbation on its neighboring elements. We implemented this approach to infer regulatory cross-talk between signaling pathways and intracellular protein transport in MCF7 cell line. Our aim is to elucidate the regulatory connection between genes constituting signaling pathways such as PI3K-Akt, Ras, Rap1, calcium, JAK-STAT, EFGR and FGFR signaling and intracellular protein transport mechanism in MCF7 cell line. We focus on PI3k-Akt signaling and Ras pathway, to highlight some of their mutual key regulatory features. In our results, we find some interesting regulatory components of PI3k-AKT signaling with respect to Ras pathway as well as intracellular protein transport mechanism. From the literature, it is known that development of resistance to cancer therapy is an important clinical problem [30]. Inactivation of apoptotic programme leads to drug resistance in tumor cells. This resistance is mainly supported by PI3K-Akt signaling and hence this signaling contributes to the resistance of cancer cell [59, 60]. As it is known that Ras and calcium signaling activate the PI3K-Akt signaling in a cell, targeting the upstream and downstream signaling pathways with respect PI3K-Akt signaling is a feasible approach to procrastinate resistance in cancer cells. In future, we will hopefully extend this work and develop a methodology as well as computational integrated platform to construct an interaction network from perturbation data not only from one cell line but simultaneously from multiple tissue samples/cell lines, for the comparative analysis of putative regulatory interactions among genes in different experimental conditions.

## Additional files

Additional file 1 Perturbed gene list. Complete list of perturbed genes. (ZIP 11 kb)

Additional file 2 DAVID Enrichment analysis 591 genes. Gene ontology and pathway enrichment analysis of the complete list of perturbed genes. (ZIP 203 kb)

Additional file 3 Node statistics table. Topological parameters of each nodes in the network. (ZIP 50 kb)

Additional file 4 Network enrichment. Gene Ontology and Pathways enrichment analysis results for the complete regulatory interaction network from 591 gene perturbation expression data. (ZIP 41 kb)

Additional file 5 Network functional modules enrichment. Module wise functional and pathways enrichment analysis of the network. (ZIP 131 kb)

Additional file 6 Enrichment terms for gene cross-talks. All regulatory interactions and enriched pathways for genes involved in cross-talk between intracellular protein transport and signaling pathway. (ZIP 100 kb)
